# Markers of collagen synthesis and degradation are increased in serum in severe sepsis: a longitudinal study of 44 patients

**DOI:** 10.1186/cc7780

**Published:** 2009-04-09

**Authors:** Fiia Gäddnäs, Marjo Koskela, Vesa Koivukangas, Juha Risteli, Aarne Oikarinen, Jouko Laurila, Juha Saarnio, Tero Ala-Kokko

**Affiliations:** 1 Department of Anesthesiology, Division of Intensive Care, Oulu University Hospital, FI-90029, Oulu, Finland; 2 Department of Surgery, Oulu University Hospital, FI-90029, Oulu, Finland; 3 Department of Clinical Chemistry, Oulu University Hospital, FI-90029, Oulu, Finland; 4 Department of Dermatology, Oulu University Hospital, FI-90029, Oulu, Finland

## Abstract

**Introduction:**

Sepsis-related multiple organ dysfunction is a common cause of death in the intensive care unit. The effect of sepsis on markers of tissue repair is only partly understood. The aim of this study was to measure markers of collagen synthesis and degradation during sepsis and investigate the association with disease severity and outcome.

**Methods:**

Forty-four patients with severe sepsis participated in the study and 15 volunteers acted as controls. Blood samples were collected for 10 days after the first sepsis-induced organ dysfunction and after three and six months. Procollagen type I and III aminoterminal propeptides (PINP and PIIINP) and cross-linked telopeptides of type I collagen (ICTP) were measured.

**Results:**

The PIIINP concentration was elevated in the septic patients (8.8 ug/L, 25th to 75th percentile = 6.8 to 26.0) when compared with controls (3.0 ug/L, 25th to 75th percentile = 2.7 to 3.3; *P *< 0.001) on day one. Maximum serum PIIINP concentrations during sepsis were higher in non-survivors compared with survivors (26.1 ug/L, 25th to 75th percentile = 18.7 to 84.3; vs. 15.1 ug/L, 25th to 75th percentile = 9.6 to 25.5; *P *= 0.033) and in multiple organ failure (MOF) compared with multiple organ dysfunction syndrome (MODS) (24.2 ug/L, 25th to 75th percentile = 13.4 to 48.2; vs. 8.9 ug/L, 25th to 75th percentile = 7.4 to 19.4; *P *= 0.002). Although the PINP values of the septic patients remained within the laboratory reference values, patients with MOF had higher values than patients with MODS (79.8, 25th to 75th percentile = 44.1 to 150.0; vs.40.4, 25th to 75th percentile = 23.6 to 99.3; *P *= 0.007). Day one ICTP levels were elevated in septic patients compared with the controls (19.4 ug/L, 25th to 75th percentile = 12.0 to 29.8; vs. 4.1 ug/L, 25th to 75th percentile = 3.4 to 5.0; *P *< 0.001).

**Conclusions:**

Markers of collagen metabolism are increased in patients with severe sepsis and can be investigated further as markers of disease severity and outcome.

## Introduction

The host response in sepsis is a dynamic process activating the pathways of coagulation, inflammation and tissue repair. When the response becomes overwhelming, it leads to multiple organ failure (MOF) and death [1-3]. Disturbed connective tissue metabolism is the key element in complications of inflammatory disease, so it was of interest to determine whether high systemic inflammation in sepsis has any effect and whether the level of connective tissue metabolism reflects disease severity and outcome.

Fibroblasts synthesise a wide array of extracellular matrix proteins, predominantly type I and III collagens, which provide structural support to the organs [4,5]. The aim of this process is to maintain tissue integrity in a steady state and restore the integrity of the organ after injury. Prolonged inflammatory response may lead to persistent or progressive fibrosis impairing the function of an organ. Collagen synthesis has been shown to be pathologically increased, not only in wound keloids and wound infections, but also in acute respiratory distress syndrome (ARDS), chronic liver diseases, myocardial infarction and kidney diseases [4,6-10]. Indeed, it has been suggested that progressive fibrosis is a central mechanism of organ failure, which is related to the host's inflammatory responses and subsequent fibroblast response [11].

In the course of collagen biosynthesis, procollagen-derived peptides are deposited in the extracellular matrix and released into the circulation. Aminoterminal propeptides are cleaved from procollagens in a one to one proportion and thus reflect the synthesis of collagen. Increased serum levels of procollagen type III propeptide have been found in severely injured patients and have been associated with MOF and death [12]. Additionally, procollagen III propeptide levels in plasma and bronchoalveolar lavage fluid from patients with ARDS are increased in early phases and are related to disease progression, multiple organ dysfunction and death [7,13].

Cross-linked type I collagen telopeptides (ICTP) were assessed as markers of collagen I degradation. Previously, Wenisch and colleagues have reported elevated ICTP levels in Gram-negative septicaemia [14].

As fibrosing activity, measured by synthesis and degradation of collagen, seems to have an important role in inflammatory processes, we hypothesised that procollagen propeptide serum levels have a prognostic value in MOF and death subsequent to severe sepsis. The collagen metabolism through the period of sepsis in humans has not been profoundly studied before.

## Materials and methods

### Patients

The study was conducted in Oulu University Hospital, Finland, which is a 900-bed tertiary-level teaching hospital. In this prospective observational study all the patients admitted to the 12-bed mixed-type adult intensive care unit (ICU) during the period from May 2005 to December 2006 were screened for eligibility for the study. The study protocol was approved by the hospital's ethics committee and all the patients or their next of kin gave written consent for inclusion in the study. The patients were treated according to the normal ICU protocol and severe sepsis guidelines [15], including steroid supplementation in septic shock. Severe sepsis and septic shock were defined according to the American College of Chest Physicians/Society of Critical Care Medicine criteria [3].

Exclusion criteria included age under 18 years, a bleeding disorder, immunosuppressant therapy, surgery not related to sepsis, surgery during the preceding six months, malignancy, chronic hepatic failure, chronic renal failure and steroid therapy not related to sepsis.

A patient entered the study when the diagnosis of severe sepsis had been confirmed and the patient or his or her next of kin had given informed consent for the study. If the time window of 48 hours from the fulfilment of the first organ dysfunction criterion was exceeded, a patient was no longer considered to be an eligible candidate for the study. Sampling was started immediately on study admission. Fifteen healthy Caucasian sex- and age-matched volunteers were used as controls (seven male and eight female). This is a part of a larger study investigating wound healing and collagen metabolism in sepsis.

### Measurements

The following information was collected from all the study patients: age, sex, type of ICU admission (medical or surgical), reason for ICU admission, focus of infection, severity of the disease on admission as assessed by acute physiology and chronic health evaluation (APACHE) II, evolution of daily organ dysfunctions assessed by daily sequential organ failure assessment (SOFA) scores and presence of chronic underlying diseases. The length of the ICU and hospital stays, as well as the ICU, hospital and 30-day mortalities, were recorded. Organ dysfunction was defined on a daily basis as an organ specific SOFA score of one to two and organ failure as a SOFA score of three to four. A patient was defined to have MOF if the daily SOFA scores of two or more organ systems were three to four on one or more days during the study period. Additively, multiple organ dysfunction syndrome (MODS) was defined as daily SOFA scores of one to two in two or more organ systems on one or more days [16,17].

### Blood samples and collagen analysis

First blood samples for procollagen types I and III aminoterminal propeptides (PINP, PIIINP) and ICTP were obtained immediately after study admission. The blood samples were collected at six-hour intervals up to 48 hours and thereafter once a day for 10 days. If a patient died or was discharged from the hospital, the follow-up was discontinued earlier. Blood samples were collected once in the control group. After centrifugation, the serum was stored at -70°C. PINP, PIIINP and ICTP were analysed using radioimmunological assays (Orion diagnostica, Espoo, Finland). Reference values are published elsewhere [18]. The coefficients of variation (CV) of the ICTP method were between 3 and 8% for a wide range of concentrations. For serum intact PINP assay, the inter- and intra-assay of CVs were 3.1 to 9.3% for values within the reference intervals. For serum PIIINP assay, inter- and intra-assay of CVs were 3.0 to 7.2% for values ranging from 2.7 to 12.2 μg/L.

### Statistical analysis

The data was analysed with SPSS (version 15.0, SPSS Inc., Chigaco, IL, USA). Unless otherwise stated, summary statistics are expressed as medians with 25th to 75th percentiles. Categorical variables were analysed with Pearson's chi-squared test or Fisher's exact test. The Mann-Whitney U test was used to analyse the differences between two groups. The PIIINP, PINP and ICTP values of the septic patients and controls on day one were compared. In addition, the maximum PIIINP, PINP and ICTP values of the surgical and medical, survivors and non-survivors, and MODS and MOF patients during the first 10 days of the study were compared. Also, patients that received cortisone therapy were compared with those who did not. The predictive value of PIIINP, PINP and ICTP on the organ failures was measured by using the receiving operating characteristics (ROC) curve analysis. The ROC curve analysis measures post-test probability. The area under ROC curves of one has the best discriminative value and the area less than 0.5 has no discriminative value. The correlations were tested with Spearman's rank order. Two-tailed *P *values are reported and differences were considered significant at *P *< 0.05. Readers should treat the *P *values with caution, because several comparisons are made, and no *P *value correction coefficient method is used. Power analysis for the study could not be conducted before the study because of a lack of previous studies on collagen turnover in severe sepsis.

## Results

### Patients

A total of 1361 patients admitted to the ICU over a period of 1.5 years were screened for eligibility. Of those, 238 admitted adult patients met the inclusion criteria and 172 of them were excluded. Of the remaining patients consent was obtained from 44 patients (29 male, 15 female) and 22 patients or their next of kin refused consent or could not be reached within 48 hours. The control group consisted of seven females and eight males. The median age of the controls was 60 years (25th to 75th percentile = 56 to 68).

There were no major differences in the patient characteristics between the surgical and medical admissions (Table 1). The median age of the whole study population was 63 years (25th to 75th percentile = 56 to 71). The median APACHE II score at the time of admission was 26 (25th to 75th percentile = 22 to 30). The most common location of infection in the surgical group was intra-abdominal (15 of 25). In the medical group, infections in the lungs were most abundant (13 of 19).

**Table 1:** Characteristics of the study patients according to type of ICU admission

	Surgical patients (25)	Medical patients (19)	*P *value	All (n = 44)
Age, years	63 (57 to 68)	63 (56 to 74)	0.9	63 (56 to 71)
Body mass index, kg/m^2^	26 (24 to 32)	26 (24 to 30)	0.8	26 (24 to 32)
Male sex	15	14	0.3	29 (66%)
Chronic diseases				
- Ischaemic heart disease	2	7		9 (20%)
- Arteriosclerosis obliterans	2	2		4 (9%)
- Diabetes	5	5		10 (23%)
- Chronic obstructive pulmonary disease	1	4		5 (11%)
- Asthma	2	2		4 (9%)
- Inflammatory disease (inflammatory bowel disease, vasculitis or rheumatoid diseases.)	0	0		0
Focus of infection				
-Lungs	5	13		18
-Intra-abdominal	14	2		16
-Urinary	1	0		1
-Primary blood	2	1		3
-Other	3	3		6
Positive blood culture	8	4	0.8	13
Hydrocortisone therapy	19	13	0.6	32 (73%)
Noradrenaline	22	16	1.0	38 (86%)
-maximum rate, μg/kg/minute	0.44 (0.29 to 1)	0.24(0.12 to 1.14)	0.3	0.42(0.19 to 1)
Adrenaline	0	1	0.4	1 (2%)
Vasopressin and analogues	3	3	1.0	6 (14%)
Activated protein C	2	4	0.4	6 (14%)
Length of stay at the intensive care unit	7 (4 to 12)	5 (4 to 12)	0.7	7 (4 to 12)
APACHE II	24 (23 to 29)	26 (22 to 30)	0.7	26 (22 to 30)
Organ failure	18	12	0.5	30 (68%)
SOFA	8 (6 to 11)	8 (7 to 11)	0.9	8 (6 to 12)
Lung spesific SOFA score even once three to four	16	10	0.4	26
Mortality during the stay at the intensive care unit	6	3	0.5	9 (21%)
Mortality (30-days)	7	4	0.6	11(25%)

Data is expressed as medians and 25th to 75th percentile or with frequencies and percentages.

APACHE II = acute physiology and chronic health evaluation II; SOFA = sequential organ failure assessment.

The blood culture was positive in 13 cases and pathogens included *Escherichia coli *(n = 3), *Streptococcus pneumoniae *(n = 1), *Klebsiella pneumoniae *(n = 1), *Klebsiella oxytoca *(n = 2), *Staphylococcus aureus *(n = 1), *Bacteroideus fragilis *(n = 3), *Clostridium paraputrificum *(n = 1), *Haemophilus influenzae *(n = 1) and *Proteus mirabilis *(n = 1).

Sixty-eight percent of cases developed MOF. Mortality over 30 days was 25%. The majority of patients (73%) received hydrocortisone treatment for septic shock refractory to noradrenaline. Noradrenaline support was needed in 86% of cases and the medium of maximum rate was 0.42 μg/kg/minute (25th to 75th percentile = 0.19 to 1.0). Need for noradrenaline support lasted for a median time of 62 hours (25th to 75th percentile = 27 to 147). One of the patients needed adrenaline for septic shock. Vasopressin or its analogues were used in six patients and activated protein C in six patients.

### Markers of collagen synthesis

#### PIIINP

**Figure 1:**
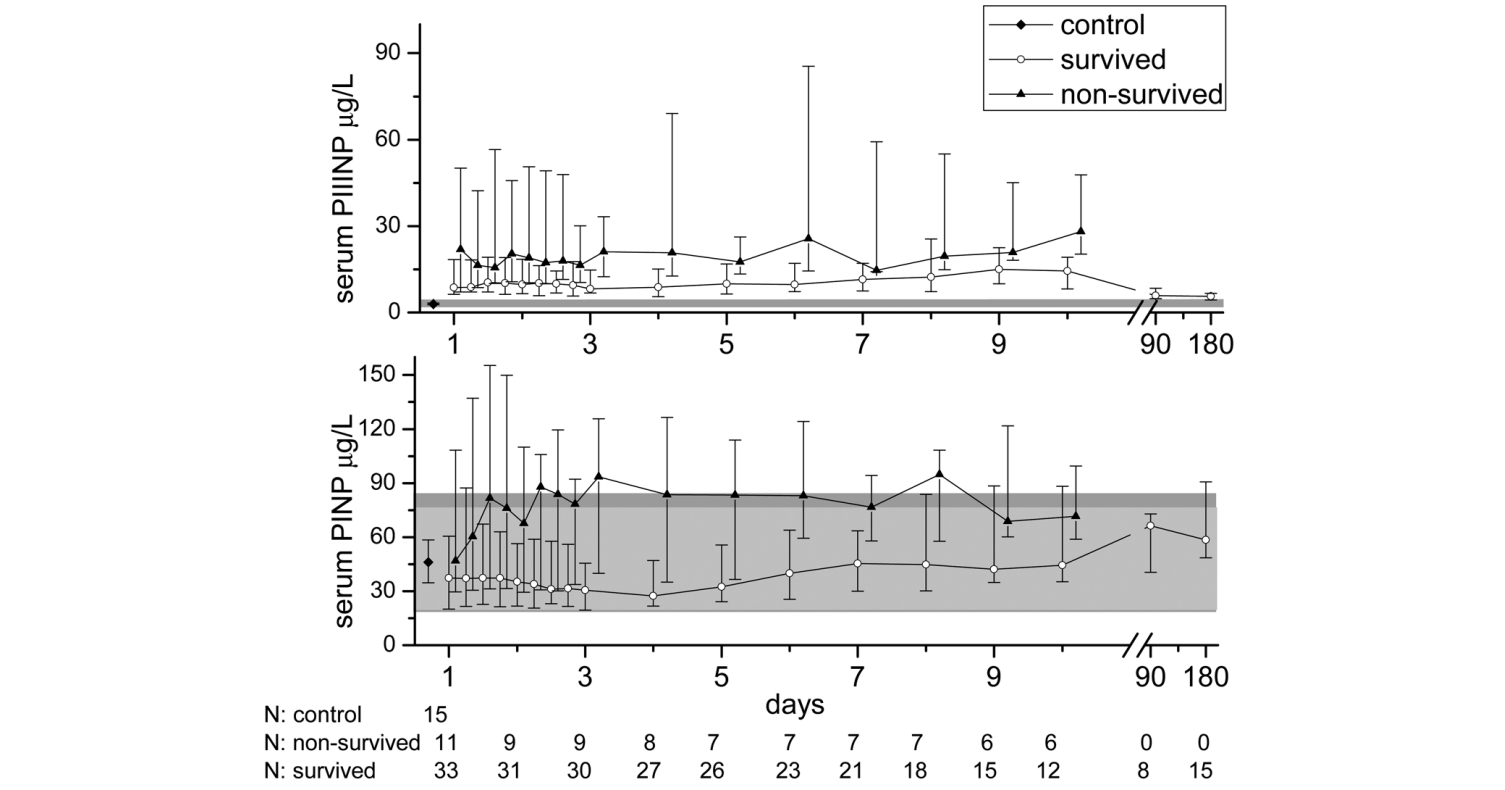
Serum procollagen I and III aminoterminal propeptide concentrations in surviving and non-survived sepsis patients during the 10-day follow up and at three and six months. The symbols mark the median values and the vertical lines stand for ranges from 25th to 75th percentile. The laboratory reference values are presented as a solid grey area in the background. Reference values for serum procollagen III aminoterminal propeptide (PIIINP) are the same for both males and females (1.7 to 4.2 μg/L). For serum procollagen I aminoterminal propeptide (PINP) the reference area for females (19 to 84 μg/L) is slightly broader than for males (20 to 76 μg/L) and is presented in darker grey.

On the first day, median PIIINP concentration was higher in septic patients (8.8 μg/L, 25th to 75th percentile = 6.8 to 26.0) compared with controls (3.0 μg/L, 25th to 75th percentile = 2.7 to 3.3; *P *< 0.001). Furthermore, the median of minimum PIIINP values of the patients over the 10-day period after the first organ failure exceeded the median PIIINP value of controls (7.2 μg/L, 25th to 75th percentile = 4.9 to 10.9; vs. 3.0 μg/L, 25th to 75th percentile = 2.7 to 3.3; *P *< 0.001). There was no significant difference in the maximum PIIINP values between the surgical sepsis patients compared with the medical patients (21.1 μg/L, 25th to 75th percentile = 13.0 to 48.2; vs. 15.1 μg/L, 25th to 75th percentile = 8.1 to 25.8; *P *= 0.159). The maximum serum PIIINP concentrations were significantly higher in nonsurvivors compared with survivors (26.1 μg/L, 25th to 75th percentile = 18.7 to 84.3; vs. 15.1 μg/L, 25th to 75th percentile = 9.6 to 25.5; *P *= 0.033), as well as in MOF compared with MODS (24.2 μg/L, 25th to 75th percentile = 13.4 to 48.2; vs. 8.9 μg/L; 25th to 75th percentile = 7.4 to 19.4; *P *= 0.002). At three and six months the surviving patients still had slightly elevated values when compared with laboratory reference values (Figure 1).

#### PINP

**Figure 2:**
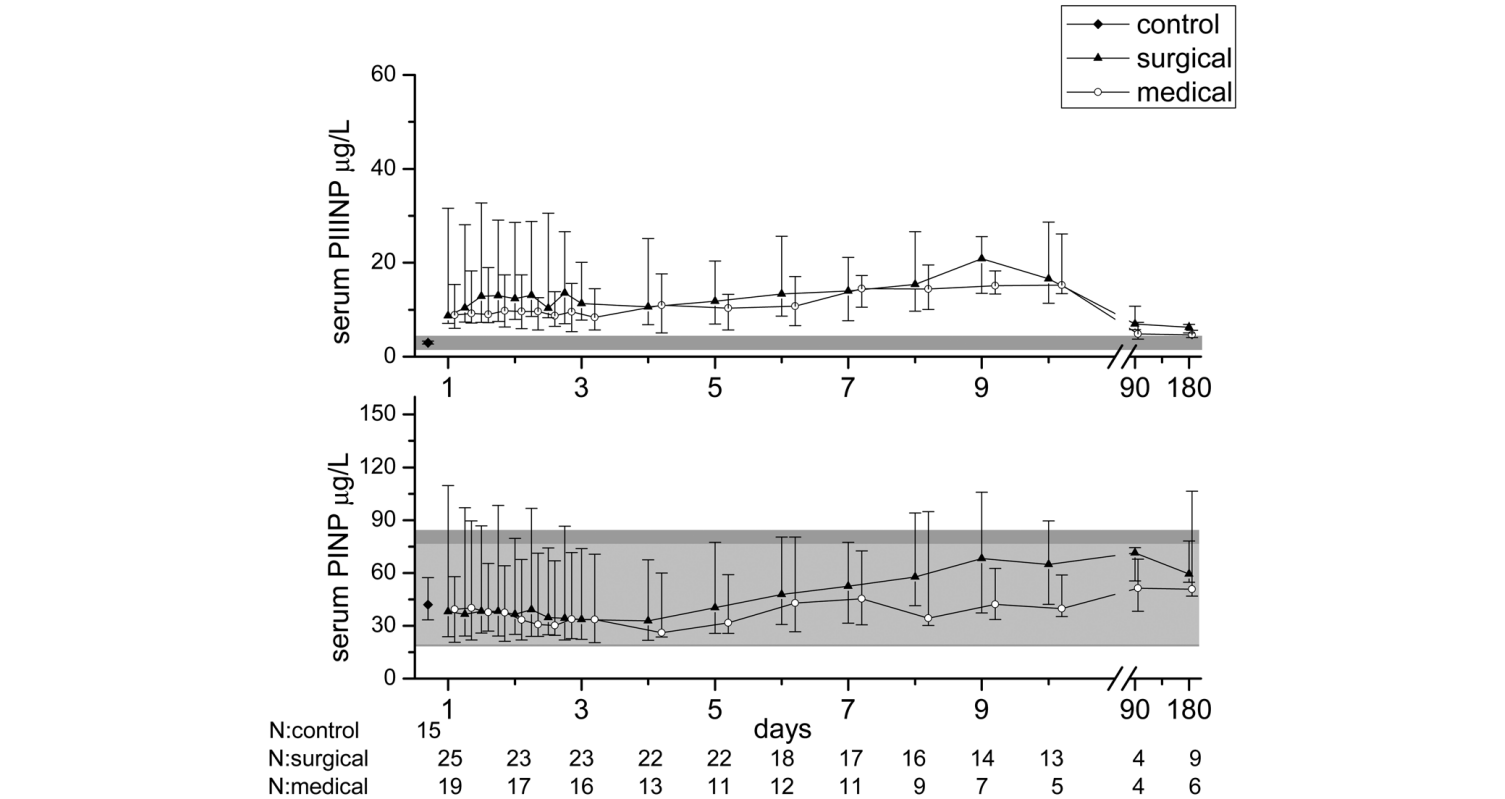
Serum procollagen I and III aminoterminal propeptide concentrations in surgical and medical groups of sepsis patients during the 10-day follow up and at three and six months. The symbols mark the median values and the vertical lines stand for ranges from 25th to 75th percentile. The laboratory reference values are presented as a solid grey area in the background. Reference values for serum procollagen III aminoterminal propeptide (PIIINP) are the same for both males and females (1.7 to 4.2 μg/L). For serum procollagen I aminoterminal propeptide (PINP) the reference area for females (19 to 84 μg/L) is slightly broader than for males (20 to 76 μg/L) and is presented in darker grey.

PINP concentrations did not differ between septic patients (38.2 μg/L, 25th to 75th percentile = 20.5 to 83.7) and controls (46.1 μg/L, 25th to 75th percentile = 34.7 to 58.4; *P *= 0.513) at the beginning of the follow-up. The maximum PINP value of the septic patients over the whole 10-day study period tended to be higher than the control value (64.0 μg/L, 25th to 75th percentile = 39.3 to 119.7; *P *= 0.054), whereas the minimum was lower (24.1 μg/L, 25th to 75th percentile = 18.5 to 39.1; *P *= 0.004). Within the first four days of the study, there was a reduction in PINP values in all septic patients. Thereafter an increase was seen, and it was more pronounced in the surgical group (Figure 2). There was no difference in the maximum PINP values between surgical and medical patients (64.9 μg/L, 25th to 75th percentile = 38.5 to 120.9; vs. 50.5 μg/L, 25th to 75th percentile = 39.8 to 114.5; *P *= 0.54) or between non-survivors and survivors (118.5 μg/L, 25th to 75th percentile = 52.4 to 190.5; vs.52.4 μg/L, 25th to 75th percentile = 38.5 to 109.7; *P *= 0.065). Although the PINP values of the septic patients remained within the laboratory reference values, the patients with MOF had higher values than patients with MODS (79.8, 25th to 75th percentile = 44.1 to 150.0; vs.40.4, 25th to 75th percentile = 23.6 to 99.3; *P *= 0.007).

### Hydrocortisone therapy

**Figure 3:**
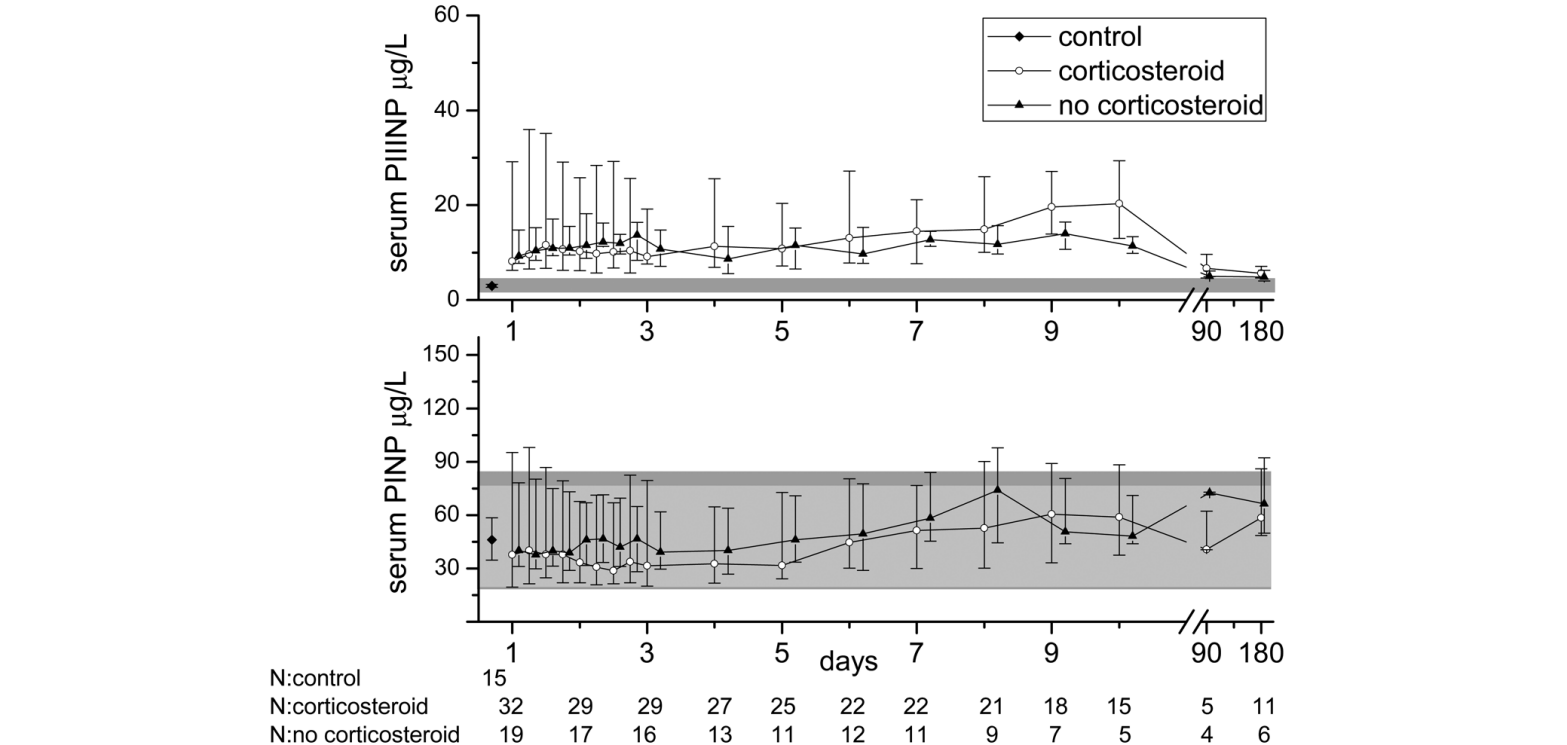
Serum procollagen I and III aminoterminal propeptide concentrations in sepsis patients during the 10-day follow up and at three and six months according to whether they received hydrocortisone supplementation or not. The symbols mark the median values and the vertical lines stand for ranges from 25th to 75th percentile. The laboratory reference values are presented as a solid grey area in the background. Reference values for serum procollagen III aminoterminal propeptide (PIIINP) are the same for both males and females (1.7 to 4.2 μg/L). For serum procollagen I aminoterminal propeptide (PINP) the reference area for females (19 to 84 μg/L) is slightly broader than for males (20 to 76 μg/L) and is presented in darker grey.

Twelve patients in the study population did not receive hydrocortisone therapy. The maximum values of markers of collagen synthesis and degradation did not differ between those receiving steroid treatment and those who did not (PIIINP: 19.9 μg/L, 25th to 75th percentile = 9.1 to 49.3; vs. 14.4 μg/L, 25th to 75th percentile = 11.5 to 22.1; *P *= 0.368; PINP: 64.0 μg/L, 25th to 75th percentile = 39.3 to 146.1; vs. 60.0 μg/L, 25th to 75th percentile = 40.0 to 146.1; *P *= 0.630; ICTP: 35.5 μg/L, 25th to 75th percentile = 20.8 to 57.9; vs. 26.7 μg/L, 25th to 75th percentile = 16.4 to 39.8; *P *= 0.358). After corticosteroid therapy, which most often continued for seven days, the serum concentration of collagen propeptides increased. The levels of PINP in hydrocortisone-treated patients were lower than in the controls or in those not receiving hydrocortisone for up to six days, and after that the PINP levels of all the patients increased above those of the controls. PIIINP levels were not down-regulated with hydrocortisone to the same extent (Figure 3). The patients who received corticosteroid medication were more severely ill: The median for maximal total SOFA score was 10.5 (25th to 75th percentile = 5.5 to 8.0) for the group receiving hydrocortisone and 7.5 (25th to 75th percentile = 8.0 to 10.0) for those who did not (*P *= 0.003).

### Correlations

PIIINP and PINP maximal concentrations as well as day one levels were analysed for correlations with 30-day mortality, maximal SOFA scores and maximal lactate levels. A positive correlation was found between day one PIIINP (*P *= 0.007) and maximal PIIINP (*P *< 0.004) concentration and 30-day mortality. Maximum PINP concentration correlated with 30-day mortality (*P *= 0.02), whereas day one PINP did not (*P *= 0.157). Day one and maximum levels of both markers of collagen synthesis correlated positively with the maximal total SOFA scores (*P *< 0.001 for both correlations). Positive correlation was also found between day one on the maximum PINP and PIIINP and maximum lactate level (*P *< 0.001 for both PINP and PIIINP) and PINP and PIIINP and liver and kidney-specific SOFA scores. ROC curve analysis of maximum PIIINP for liver failure shows an area under the curve (AUC) of 0.737 (95% confidence interval (CI) = 0.518 to 0.956; *P *= 0.065) and for renal failure an AUC of 0.545 (95% CI = 0.233 to 0.856; *P *= 0.798). ROC curve analysis of maximum PINP for liver failure shows an AUC of 0.750 (95% CI = 0.564 to 0.936; *P *= 0.051) and for renal failure an AUC of 0.520 (95% CI = 0.163 to 0.877; *P *= 0.907).

### ICTP, marker of collagen degradation

**Figure 4:**
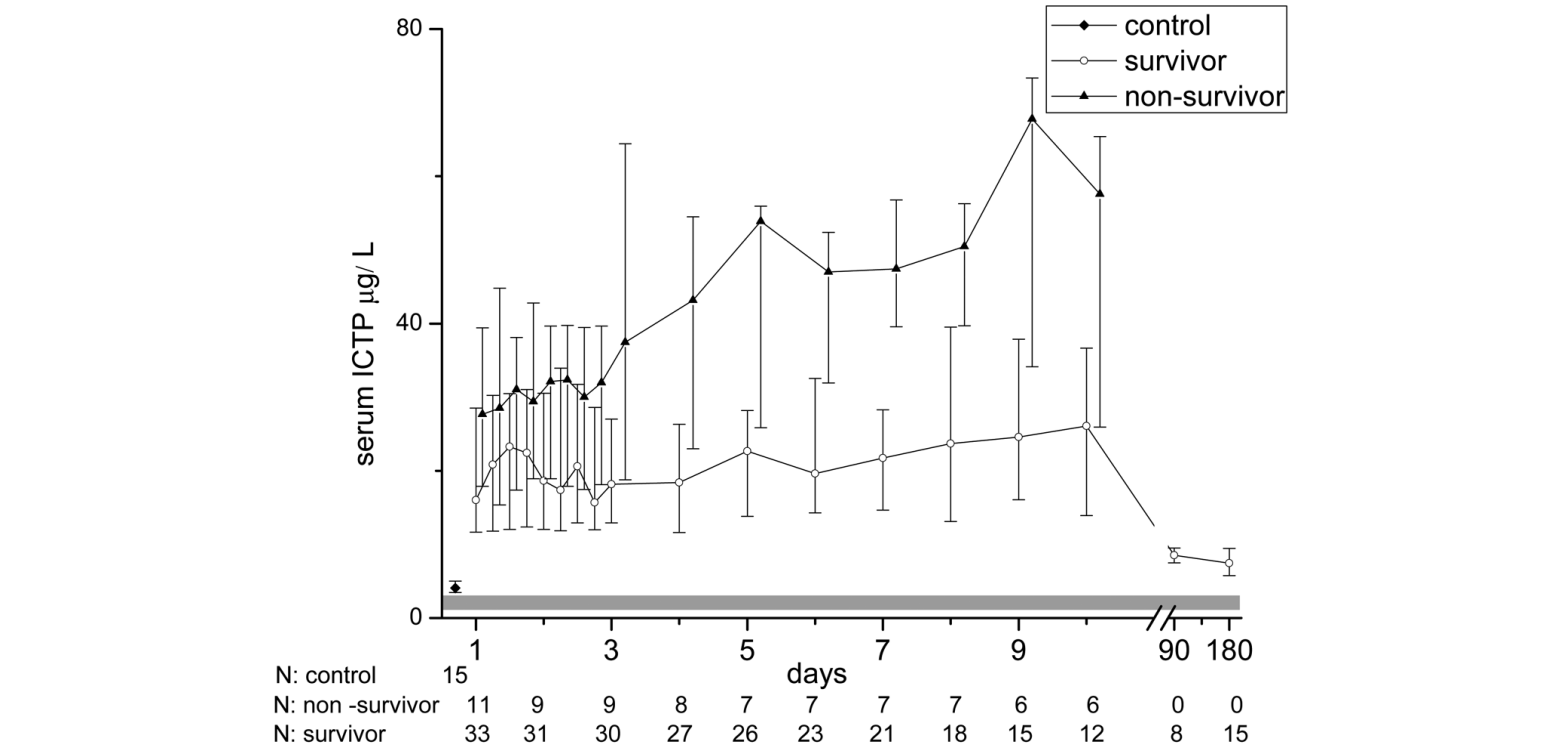
Concentrations of type I collagen cross-linked telopeptides in groups of survived and non-survived sepsis patients during the 10-day follow up and at three and six months. The laboratory reference values (1.6 to 4.6 μg/l) are presented as a solid grey area in the background. The symbols mark the median values and the vertical lines stand for ranges from 25th to 75th percentile. ICTP = type I collagen cross-linked telopeptides.

ICTP levels were higher in the septic patients compared with the controls (19.4 μg/L, 25th to 75th percentile = 12.0 to 29.8; vs. 4.1 μg/L, 25th to 75th percentile = 3.4 to 5.0; *P *< 0.001) on the first day. The maximum and minimum values over the 10-day period were clearly higher in comparison with the control value (31.3 μg/L, 25th to 75th percentile = 18.3 to 49.0; *P *< 0.001; 16.0 μg/L, 25th to 75th percentile = 10.5 to 26.5; *P *< 0.001). The surgical patients had maximum ICTP values similar to those of the medical patients (35.3 μg/L, 25th to 75th percentile = 25.3 to 56.2; vs. 27.0 μg/L, 25th to 75th percentile = 15.2 to 41.5; *P *= 0.115). Non-survivors had higher concentrations than survivors (39.0 μg/L, 25th to 75th percentile = 30.6 to 73.7; vs. 27.9 μg/L, 25th to 75th percentile = 16.0 to 44.4; *P *= 0.038), and the difference tended to increase with time (Figure 4). The same trend was found in the MOF group compared with patients with MODS (37.8 μg/L, 25th to 75th percentile = 26.3 to 66.1; vs. 6.7 μg/L, 25th to 75th percentile = 11.4 to 34.3; *P *= 0.004). The patients that received hydrocortisone therapy had no statistically significant difference in maximum ICTP value compared with those who did not receive supplementation therapy (35.5 μg/L, 25th to 75th percentile = 20.8 to 57.9; vs. 26.7 μg/L, 25th to 75th percentile = 16.4 to 39.8; *P *= 0.343).

The maximum ICTP value correlated positively with the maximum total SOFA score and maximum lactate levels (*P *= 0.000; *P *= 0.011). Also ICTP level on day one correlated with maximum total SOFA score (*P *< 0.001) and maximum lactate levels (*P *= 0.013). ROC curve analysis of maximum ICTP for liver failure shows an AUC of 0.610 (25th to 75th percentile = 0.450 to 0.769; *P *= 0.393) and for renal failure an AUC of 0.472 (25th to 75th percentile = 0.252 to 0.691; *P *= 0.871). Neither maximum nor day one ICTP correlated with 30-day mortality.

## Discussion

This is the first longitudinal study reporting serum procollagen propeptide levels in human severe sepsis. Previous studies have focused on collagen metabolism in severe trauma, ARDS or Gram-negative sepsis [7,12,14]. Increasing collagen propeptide levels (PIIINP throughout the disease process and PINP in the late phase) were associated with the development of MOF and death and they correlated with maximum lactate concentrations. All the values in survivors had returned to the normal range and were lower at three and six months than they were at the beginning of the study.

Of the different organs, collagen synthesis in lungs has been most profoundly studied in critical illness. ARDS is the most severe manifestation of acute lung injury and is also one of the most common organ failures in severe sepsis. The collagen I and III propeptides have been showed to be elevated in plasma and bronchoalveolar lavage fluid in patients with ARDS during the first days of disease and are associated with increased risk of death [7,13,19]. In our data the patients with lung specific SOFA scores of three to four had only slightly pronounced PINP, PIIINP and ICTP values (day one and the maximal values over the study period) compared with patients with less severe scores. The difference did not reach statistical significance (data not shown). Hence the increased procollagen propeptide levels observed in this study seem to be only partly due to increased synthesis and degradation of collagen in the lungs.

Waydhas and colleagues reported increased PIIINP serum concentrations in severely injured patients [12]. Similar to our findings in septic patients, serum concentrations were elevated in severely injured non-survivors and in those who developed MOF. It was noted in the study by Waydhas and colleagues that PIIINP levels correlated with increasing bilirubin levels. The procollagen propeptides are eliminated by the liver, thus the increased serum levels may result from increased synthesis or decreased uptake by liver cells [20]. The study by Waydhas and colleagues did not determine whether the increased concentrations were due to excess synthesis or diminished elimination [12].

On the other hand, in alcoholic liver fibrosis it has been shown that elevated PIIINP concentrations are caused by increased histologically confirmed fibrogenesis [21]. In our study, PINP and PIIINP did correlate with liver function implying that either synthesis or elimination by the liver in sepsis is affected. Because PINP and PIIINP are eliminated via the same liver endothelial cell receptor, the serum levels of both propeptides should have increased if the increased concentrations were solely a result of decreased elimination.

A correlation with kidney function was also observed. Small fractions of PIIINP are excreted by the kidneys [22,23]. Interestingly, increased PIIINP levels have been reported in acute renal disease, exemplifying the influence of systemic disease on collagen metabolism. Keller and colleagues reported that, compared with values in chronic renal failure, the values of PIIINP were even higher in patients with acute renal failure and MOF [8]. Furthermore, experimental data have shown that renal damage increases the release of a collagen synthesis-stimulating factor [24]. Previous data thus suggests that acute renal failure is associated with increased synthesis of type III collagen. In the present study, maximum PINP, PIIINP and ICTP levels did not have statistically significant prognostic values for liver and renal failure in the ROC analysis.

It is tempting to speculate that increased collagen propeptide levels found are at least partly due to increased synthesis and are likely to be a summation of collagen synthesis from different organs. To find out the contribution of the different organs affected, further studies are required.

ICTP is a marker of collagen degradation and is eliminated by the kidneys [20]. In a small study Wenisch and colleagues reported elevated ICTP levels in Gram-negative sepsis on day 0 and day 28 [14]. We found that serum ICTP, but not PINP, was increased in severe sepsis. Thus, the increased ICTP levels most likely indicate increased degradation of collagen type I. As type I collagen is most abundant in bone, it could be speculated that high levels of ICTP could partly be a result of immobilisation. However, increased ICTP levels most likely mirror high systemic inflammation, because the levels were highest in patients with the most severe forms of the disease. Collagens are degraded by specific matrix metalloproteinases (MMPs) produced by fibroblasts, other connective tissue cells and inflammatory cells. MMPs are induced by proinflammatory cytokines (e.g. IL-1, IL-6 and TNF). *In vitro *it has been shown that, following exposure to *S. aureus*, fibroblasts have increased MMP expression, which is associated with degradation of collagen [25].

Our study suggests that collagen turnover may be increased in severe sepsis. Over the past years, our understanding on the complexity of the host healing response in sepsis has grown: Phases of coagulation, inflammation and fibroproliferation overlap and exert regulatory control on one another. The collagen synthesis in fibroblasts is regulated by coagulation cascade proteases, proinflammatory cytokines and growth factors. Coagulation protease thrombin seems to act as fibroblast chemoattractant [26], stimulator of procollagen production [27], promoter of myofibroblast formation [28] and MMP activator [29]. Recently, a similar role of the upstream coagulation protease Xa has been acknowledged. It seems to enhance the expression of tranforming growth factor beta (TGF-β), fibroblast proliferation and differentiation to myofibroblasts, migration and fibronectin production [30]. Thus the activated coagulation in sepsis is one factor promoting the fibrogenetic response.

Of the proinflammatory cytokines TNF-α has a pivotal effect on collagen synthesis. In addition to stimulating fibroblast growth and collagen synthesis, it has been shown that TNF-α in high concentrations inhibits collagen and fibronectin production and induces collagenase synthesis [31]. Among the growth factors TGF-β deserves special attention. It is a multifunctional growth factor that regulates proliferation, differentiation of cells, protein synthesis and angiogenesis. TGF-β has been reported to act as an inducer, as well as an inhibitor, of fibroblast growth [32]. Increased fibrosis is mediated by TGF-β1 in various disease states, and progressive fibrosis has been suggested to be a common pathway to organ failure [11]. Accordingly, in ARDS it has been demonstrated that bronchoalveolar lavage fluid obtained from patients is capable of activating a human procollagen 1 promoter by means of TGF-β1 present in the bronchoalveolar lavage fluid. Furthermore, in ARDS TGF-β1 levels have been shown to be higher in non-survivors, although the result is not statistically significant [33]. Higher levels have also been reported in trauma patients developing sepsis [34]. Indeed, sepsis could be called a systemic wound with activated coagulation, inflammation and fibrogenetic response.

Other factors that can affect collagen metabolism in severe sepsis include surgery, hydrocortisone treatment and tissue hypoxia. Surgery and trauma induce the healing process and thus account for the fibroproliferative response. In a previous study, it was shown that surgery itself (and wound infection especially) increases serum procollagen concentrations [35]. In our study no differences could be found between the surgical and medical groups. The surgical group consisted of patients with trauma or those who underwent major surgical procedure requiring general anaesthesia. Minor standard ICU procedures such as tracheostomy, drainage or cannulations were also performed in the medical group and could partly have contributed to the controversial result of our study.

It is known that corticosteroid therapy reduces collagen deposition [7,36]. In our material, treatment of sepsis with steroids decreased serum PINP levels, indicating that type I collagen synthesis is decreased in the early phase (up to six days) of sepsis in patients treated with hydrocortisone. After hydrocortisone therapy, which most often lasted seven days, the PINP values were upregulated as in the group not treated with hydrocortisone. Hypoxia is a fibrotic stimulus associated with enhanced collagen synthesis and it has been shown to augment collagen prolyl 4-hydroxylase activity *in vitro *[37]. Tissue hypoxia and activation of the coagulation and inflammatory cascades play a key role in the pathogenesis of MODS. Although adequate initial resuscitation usually restores oxygen delivery at the systemic level, regional hypoxia at the organ level is a well-documented phenomenon. The mechanisms are considered to include microcirculatory disturbances, that block the oxygen supply, and mitochondrial malfunction that results in inadequate use of oxygen at the cellular level. Increased circulating lactate levels are suggestive of tissue hypoxia and are associated with a poor outcome. In our study PINP, PIIINP and ICTP correlated with maximum lactate levels. The importance of tissue hypoxia in the stimulation of collagen synthesis is also suggested by the results in patients with chronic heart failure in which relative collagen deposition in the intestinal wall was the highest in advanced cases of heart failure [38]. Furthermore, in a rat model, sepsis has been shown to induce significant increases in collagen content in hepatic and ileal interstitial tissues, which were prevented with a leucotriene antagonist [39]. Yet there is also evidence to the contrary. In a mice model of lipopolysaccharide-stimulated ARDS, hypoxia suppressed inflammation in lungs via adenosine A_2A_-receptor-mediated pathway and resulted in lower lung injury score and thickening of the alveocapillary membrane [40].

This study is limited by the fact that our study population was relatively small because this was a one-centre study and a considerable number of patients were excluded because of underlying diseases affecting collagen metabolism. Second, the controls were healthy volunteers and thus could not be matched for chronic diseases, of which arteriosclerosis, diabetes and pulmonary diseases may have altered collagen metabolism. Third, the serum markers of inflammation were not measured. The septic response is individual and patients may have entered the study in different phases of inflammation, although all of them entered within 48 hours of the first organ failure. Further studies are needed to connect the levels of collagen turnover to timely development of coagulation and inflammatory responses. Nonetheless, this study provides new *in vivo *measured information on connective tissue metabolism and its timely development in sepsis.

## Conclusions

Serum levels of PIIINP and ICTP are significantly increased in patients with severe sepsis and can be investigated further as markers of disease severity and outcome. These results imply that fibrosis may be a central mechanism in the pathogenesis of multiple organ dysfunction.

## Key messages

• Serum levels of PIIINP and ICTP are significantly increased in patients with severe sepsis and can be investigated further as markers of disease severity and outcome.

• PIIINP and ICTP values in survivors returned to the normal range and were lower at three and six months than they were at the beginning of the study.

## Abbreviations

APACHE II: acute physiology and chronic health evaluation II; ARDS: adult respiratory distress syndrome; AUC: area under the curve; CI: confidence interval; CV: coefficients of variation; ICTP: collagen I cross-linked telopeptide; ICU: Intensive care unit; IL: interleukin; MMP: matrix metalloproteinase; MODS: multiple organ dysfunction syndrome; MOF: multiple organ failure; PINP: procollagen I aminoterminal propeptide; PIIINP: procollagen III aminoterminal propeptide; ROC: receiving operating characteristics; SOFA: sequential organ failure assessment; TGF: transforming growth factor; TNF: tumour necrosis factor.

## Competing interests

The authors declare that they have no competing interests.

## Authors' contributions

All authors participated in the study design. FG participated in collecting the data, performed statistical analysis and drafted the manuscript with TA. MK participated in collecting the data. VK conceived the study and helped to draft the manuscript. AO provided the equipment for the suction blister method and helped to draft the manuscript. JR provided collagen propeptide analyses. JL helped to draft the manuscript. JS conceived the study with VK. TA performed the statistical analysis and drafted the manuscript with FG. All authors read and approved the final manuscript.

## References

[B1] Bone RC, Balk RA, Cerra FB, Dellinger RP, Fein AM, Knaus WA, Schein RM, Sibbald WJ (1992). Definitions for sepsis and organ failure and guidelines for the use of innovative therapies in sepsis. The ACCP/SCCM Consensus Conference Committee. American College of Chest Physicians/Society of Critical Care Medicine. Chest.

[B2] Hotchkiss RS, Karl IE (2003). The pathophysiology and treatment of sepsis. N Engl J Med.

[B3] Levy MM, Fink MP, Marshall JC, Abraham E, Angus D, Cook D, Cohen J, Opal SM, Vincent JL, Ramsay G, SCCM/ESICM/ACCP/ATS/SIS (2003). 2001 SCCM/ESICM/ACCP/ATS/SIS International Sepsis Definitions Conference. Crit Care Med.

[B4] Haukipuro K, Risteli L, Kairaluoma MI, Risteli J (1987). Aminoterminal propeptide of type III procollagen in healing wound in humans. Ann Surg.

[B5] Petri JB, Konig S, Haupt B, Haustein UF, Herrmann K (1997). Molecular analysis of different phases in human wound healing. Exp Dermatol.

[B6] Jensen LT, Horslev-Petersen K, Toft P, Bentsen KD, Grande P, Simonsen EE, Lorenzen I (1990). Serum aminoterminal type III procollagen peptide reflects repair after acute myocardial infarction. Circulation.

[B7] Meduri GU, Tolley EA, Chinn A, Stentz F, Postlethwaite A (1998). Procollagen types I and III aminoterminal propeptide levels during acute respiratory distress syndrome and in response to methylprednisolone treatment. Am J Respir Crit Care Med.

[B8] Keller F, Rehbein C, Schwarz A, Fleck M, Hayasaka A, Schuppan D, Offermann G, Hahn EG (1988). Increased procollagen III production in patients with kidney disease. Nephron.

[B9] Moller S, Hansen M, Hillingso J, Jensen JE, Henriksen JH (1999). Elevated carboxy terminal cross linked telopeptide of type I collagen in alcoholic cirrhosis: relation to liver and kidney function and bone metabolism. Gut.

[B10] Zhang K, Garner W, Cohen L, Rodriguez J, Phan S (1995). Increased types I and III collagen and transforming growth factor-beta 1 mRNA and protein in hypertrophic burn scar. J Invest Dermatol.

[B11] Weber KT (1997). Fibrosis, a common pathway to organ failure: angiotensin II and tissue repair. Semin Nephrol.

[B12] Waydhas C, Nast-Kolb D, Trupka A, Lenk S, Duswald KH, Schweiberer L, Jochum M (1993). Increased serum concentrations of procollagen type III peptide in severely injured patients: an indicator of fibrosing activity?. Crit Care Med.

[B13] Clark JG, Milberg JA, Steinberg KP, Hudson LD (1995). Type III procollagen peptide in the adult respiratory distress syndrome. Association of increased peptide levels in bronchoalveolar lavage fluid with increased risk for death. Ann Intern Med.

[B14] Wenisch C, Graninger W, Schonthal E, Rumpold H (1996). Increased serum concentrations of the carboxy-terminal cross-linked telopeptide of collagen type I in patients with Gram-negative septicaemia. Eur J Clin Invest.

[B15] Dellinger RP, Carlet JM, Masur H, Gerlach H, Calandra T, Cohen J, Gea-Banacloche J, Keh D, Marshall JC, Parker MM, Ramsay G, Zimmerman JL, Vincent JL, Levy MM, Surviving Sepsis Campaign Management Guidelines, Committee (2004). Surviving Sepsis Campaign guidelines for management of severe sepsis and septic shock. Crit Care Med.

[B16] Vincent JL, de Mendonca A, Cantraine F, Moreno R, Takala J, Suter PM, Sprung CL, Colardyn F, Blecher S (1998). Use of the SOFA score to assess the incidence of organ dysfunction/failure in intensive care units: results of a multicenter, prospective study. Working group on "sepsis-related problems" of the European Society of Intensive Care Medicine. Crit Care Med.

[B17] Vincent JL, Moreno R, Takala J, Willatts S, De Mendonca A, Bruining H, Reinhart CK, Suter PM, Thijs LG (1996). The SOFA (Sepsis-related Organ Failure Assessment) score to describe organ dysfunction/failure. On behalf of the Working Group on Sepsis-Related Problems of the European Society of Intensive Care Medicine. Intensive Care Med.

[B18] Risteli Juha RL, Royce PM, Steinmann B (2002). Extracellular matrix metabolites in body fluids. In Connective tissue and its heritable disorders: molecular, genetic and medical aspects.

[B19] Marshall RP, Bellingan G, Webb S, Puddicombe A, Goldsack N, McAnulty RJ, Laurent GJ (2000). Fibroproliferation occurs early in the acute respiratory distress syndrome and impacts on outcome. Am J Respir Crit Care Med.

[B20] Risteli J, Risteli L (1995). Analysing connective tissue metabolites in human serum. Biochemical, physiological and methodological aspects. J Hepatol.

[B21] Nøjgaard C, Johansen JS, Christensen E, Skovgaard LT, Price PA, Becker U (2003). Serum levels of YKL-40 and PIIINP as prognostic markers in patients with alcoholic liver disease. J Hepatol.

[B22] Bentsen KD, Boesby S, Kirkegaard P, Hansen CP, Jensen SL, Horslev-Petersen K, Lorenzen I (1988). Is the aminoterminal propeptide of type III procollagen degraded in the liver? A study of type III procollagen peptide in serum during liver transplantation in pigs. J Hepatol.

[B23] Jensen LT, Blaehr H, Andersen CB, Risteli J, Lorenzen I (1992). Metabolism of the aminoterminal propeptide of type III procollagen in cultures of human proximal tubular cells. Scand J Clin Lab Invest.

[B24] Ohyama K, Seyer JM, Raghow R, Kang AH (1990). A factor from damaged rat kidney stimulates collagen biosynthesis by mesangial cells. Biochim Biophys Acta.

[B25] Kanangat S, Postlethwaite A, Hasty K, Kang A, Smeltzer M, Appling W, Schaberg D (2006). Induction of multiple matrix metalloproteinases in human dermal and synovial fibroblasts by Staphylococcus aureus: implications in the pathogenesis of septic arthritis and other soft tissue infections. Arthritis Res Ther.

[B26] Dawes KE, Gray AJ, Laurent GJ (1993). Thrombin stimulates fibroblast chemotaxis and replication. Eur J Cell Biol.

[B27] Chambers RC, Dabbagh K, McAnulty RJ, Gray AJ, Blanc-Brude OP, Laurent GJ (1998). Thrombin stimulates fibroblast procollagen production via proteolytic activation of protease-activated receptor 1. Biochem J.

[B28] Bogatkevich GS, Tourkina E, Silver RM, Ludwicka-Bradley A (2001). Thrombin differentiates normal lung fibroblasts to a myofibroblast phenotype via the proteolytically activated receptor-1 and a protein kinase C-dependent pathway. J Biol Chem.

[B29] Duhamel-Clerin E, Orvain C, Lanza F, Cazenave JP, Klein-Soyer C (1997). Thrombin receptor-mediated increase of two matrix metalloproteinases, MMP-1 and MMP-3, in human endothelial cells. Arterioscler Thromb Vasc Biol.

[B30] Borensztajn K, Stiekema J, Nijmeijer S, Reitsma PH, Peppelenbosch MP, Spek CA (2008). Factor Xa stimulates proinflammatory and profibrotic responses in fibroblasts via protease-activated receptor-2 activation. Am J Pathol.

[B31] Maish GO, Shumate ML, Ehrlich HP, Cooney RN (1998). Tumor necrosis factor binding protein improves incisional wound healing in sepsis. J Surg Res.

[B32] Thornton SC, Por SB, Walsh BJ, Penny R, Breit SN (1990). Interaction of immune and connective tissue cells: I. The effect of lymphokines and monokines on fibroblast growth. J Leukoc Biol.

[B33] Budinger GR, Chandel NS, Donnelly HK, Eisenbart J, Oberoi M, Jain M (2005). Active transforming growth factor-beta1 activates the procollagen I promoter in patients with acute lung injury. Intensive Care Med.

[B34] Laun RA, Schroder O, Schoppnies M, Roher HD, Ekkernkamp A, Schulte KM (2003). Transforming growth factor-beta1 and major trauma: time-dependent association with hepatic and renal insufficiency. Shock.

[B35] Haukipuro K, Risteli L, Kairaluoma MI, Risteli J (1990). Aminoterminal propeptide of type III procollagen in serum during wound healing in human beings. Surgery.

[B36] Oikarinen A, Autio P, Vuori J, Vaananen K, Risteli L, Kiistala U, Risteli J (1992). Systemic glucocorticoid treatment decreases serum concentrations of carboxyterminal propeptide of type I procollagen and aminoterminal propeptide of type III procollagen. Br J Dermatol.

[B37] Fahling M, Mrowka R, Steege A, Nebrich G, Perlewitz A, Persson PB, Thiele BJ (2006). Translational control of collagen prolyl 4-hydroxylase-alpha(I) gene expression under hypoxia. J Biol Chem.

[B38] Arutyunov GP, Kostyukevich OI, Serov RA, Rylova NV, Bylova NA (2008). Collagen accumulation and dysfunctional mucosal barrier of the small intestine in patients with chronic heart failure. Int J Cardiol.

[B39] Sener G, Sehirli O, Cetinel S, Ercan F, Yuksel M, Gedik N, Yegen BC (2005). Amelioration of sepsis-induced hepatic and ileal injury in rats by the leukotriene receptor blocker montelukast. Prostaglandins Leukot Essent Fatty Acids.

[B40] Thiel M, Chouker A, Ohta A, Jackson E, Caldwell C, Smith P, Lukashev D, Bittmann I, Sitkovsky MV (2005). Oxygenation inhibits the physiological tissue-protecting mechanism and thereby exacerbates acute inflammatory lung injury. PLoS Biol.

